# Beating-Heart Pulmonary Valve Replacement for Isolated Monocuspid Valve

**DOI:** 10.1016/j.jaccas.2025.105536

**Published:** 2025-10-22

**Authors:** Mohammad Abbasi, Abdul Rahman Alizada, Navid Abbasiyan Fallahi

**Affiliations:** a Mashhad University of Medical Sciences, Mashhad, Iran; b Department of Cardiac Surgery, Razavi Hospital, Mashhad, Iran; c Student Research Committee, School of Nursing and Midwifery, Semnan University of Medical Sciences, Semnan, Iran

**Keywords:** cardioplegia, cardiopulmonary bypass, case report, congenital heart defects, pulmonary artery aneurysm, right ventricular dilation

## Abstract

**Background:**

A monocuspid pulmonary valve is a rare cardiac anomaly that is most commonly seen in patients with other congenital heart defects. Its isolated occurrence is extremely rare.

**Case Summary:**

A 20-year-old woman with a history of congenital heart disease presented with exertional dyspnea and generalized weakness. Imaging revealed severe right ventricular dilation and significant enlargement of the main pulmonary artery. Owing to the high risk of aneurysm rupture, she underwent aneurysmectomy, aneurysmorrhaphy, and pulmonary valve replacement (PVR). The heart was maintained in a beating state throughout the procedure, including during cardiopulmonary bypass without cardioplegic arrest.

**Discussion:**

This case demonstrates the feasibility of performing PVR and aneurysm repair using the beating-heart technique.

**Conclusions:**

Monocuspid pulmonary valve can occur in isolation and may cause progressive pulmonary valve insufficiency and aneurysmal dilation. Beating-heart PVR is a novel and less invasive surgical approach compared with traditional methods.

## Graphical Abstract

**Figure:**
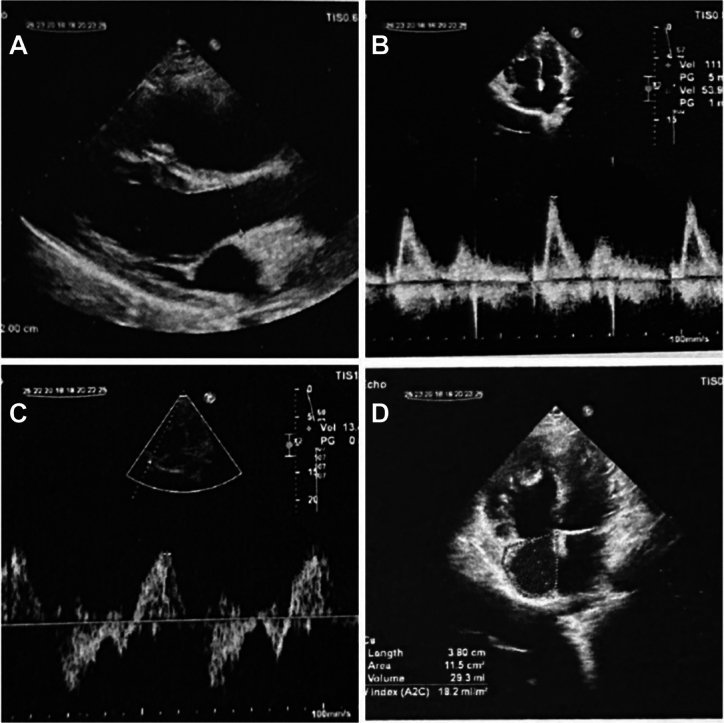

The incidence of congenital heart disease varies between 5 and 8 per 1,000 live births.^1^ Pulmonary valve regurgitation is a rare cardiac anomaly that is most commonly seen in patients with a history of congenital heart surgeries, such as tetralogy of Fallot repair. In contrast, severe congenital pulmonary valve regurgitation is extremely rare. In a small number of these patients, the regurgitation results from abnormal valve morphology, such as a monocuspid pulmonary valve. These patients may remain clinically silent until adulthood, unless progressive complications such as exertional dyspnea develop. Additionally, pulmonary artery aneurysm (PAA) is a disorder of varying etiology and should be diagnosed early for appropriate interventions.^2^ PAA is a rare condition treated in various ways depending on its location and size.^3^ Pulmonary valve replacement (PVR) and aneurysmorrhaphy are usually performed under cardiopulmonary bypass (CPB) because of the need for a bloodless surgical field, optimal visualization, and precise reconstruction of the pulmonary valve and aneurysmal segments. Although beating-heart technique PVR is not yet widely practiced, recent evidence suggests that in selected cases and with the use of advanced techniques such as hybrid approaches, valve replacement has been successfully performed with this technique.

**Take-Home Messages**

• Early diagnosis of congenital pulmonary valve anomalies can prevent life-threatening complications such as aneurysm rupture.
• In PVR, the on-pump beating-heart technique without cardioplegic arrest may reduce surgical complications compared with traditional methods.

We report a rare case of isolated monocuspid pulmonary valve regurgitation associated with a PAA, which was successfully treated with beating-heart PVR. This approach demonstrates the feasibility and safety of performing these complex surgical procedures without the need for cardioplegic arrest during CPB, while preserving cardiac function and reducing postoperative complications.

## History of presentation

A 20-year-old woman with a history of congenital heart disease had been under regular medical supervision. She presented with progressive dyspnea over the past 3 months. She visited her physician after having experienced chest pain, cough, dizziness, and generalized weakness.

## Past medical history

Owing to the absence of significant clinical manifestations and the lack of associated congenital cardiac anomalies, the patient had not undergone any prior cardiac surgery. She was under routine medical follow-up.

## Investigations

Electrocardiogram showed a sinus rhythm with signs of right ventricular (RV) hypertrophy and right axis deviation. On cardiac auscultation, the pulmonary component of the second heart sound was absent and instead, a loud to-and-fro murmur was heard in the pulmonary area, strongly suggestive of severe pulmonary valve insufficiency and abnormal valve function. On chest x-ray, there was moderate cardiomegaly, and an aneurysmal dilation of the main pulmonary artery was noted.

**Figure 1:**
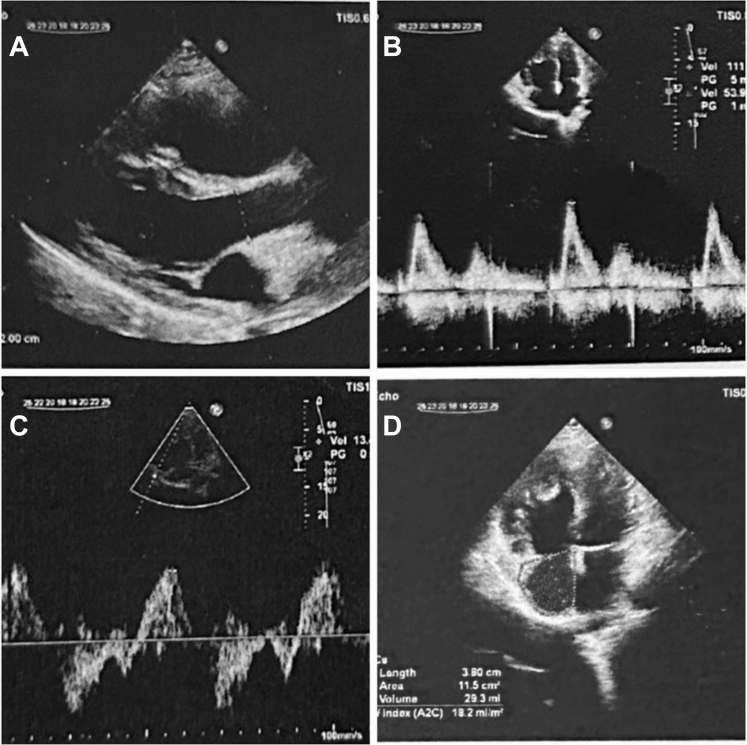
Preoperative Echocardiographic Assessment (A) Parasternal short-axis view of the right ventricular outflow tract showing abnormal pulmonary valve. (B) Continuous-wave Doppler across the pulmonary valve showing severe pulmonary regurgitation. (C) Tissue Doppler imaging at the tricuspid annulus assessing right ventricular systolic function. (D) Apical 4-chamber view showing right ventricular and right atrial dilation.

To assess pulmonary valve regurgitation and RV function, preoperative echocardiographic images were reviewed using tissue Doppler imaging. The evaluation showed severe dilation of the RV and right atrium, along with reduced systolic function. A dysplastic monocuspid pulmonary valve was observed, with severe free pulmonary regurgitation and no significant stenosis. An aneurysmal dilation of the main pulmonary artery was noted (Table 1, Figure 1).

**Table 1:** Quantitative Echocardiographic Parameters

	Measured Value	Reference	Interpretation
RV basal diameter (A4C view)	46 mm	≤42 mm	Dilated
RV midcavity diameter (A4C view)	39 mm	≤35 mm	Dilated
RV longitudinal length	91 mm	≤86 mm	Dilated
TAPSE	6.3 mm	≥17 mm	Severely reduced
FAC	28%	≥35%	Reduced
S′ (TDI at tricuspid annulus)	13.5 cm/s	≥9.5 cm/s	Preserved
RVOT diameter	41 mm	≤30 mm	Dilated
RA area	20 cm^2^	18 cm^2^	Enlarged
PASP (from TR jet)	27 mm Hg	<35 mm Hg	Normal/mildly elevated

A4C = apical 4-chamber; FAC = fractional area change; PASP = pulmonary artery systolic pressure; RA = right atrium; RV = right ventricle; RVOT = right ventricular outflow tract; TAPSE = tricuspid annular plane systolic excursion; TDI = tissue Doppler imaging; TR = tricuspid regurgitation.

**Figure 2:**
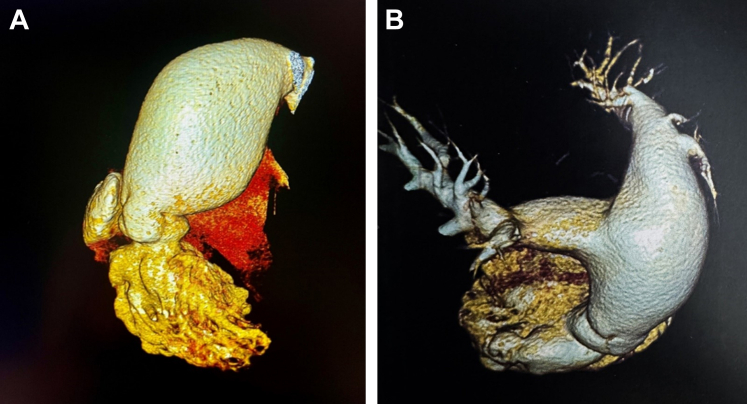
Computed Tomography Angiography of the Pulmonary Artery (A and B) Significant enlargement of the main pulmonary artery is evident. Although the right pulmonary artery and left pulmonary artery appeared normal at the hilum, their diameters at the ostium suggest aneurysmal changes. Clear dilation of the main pulmonary trunk is also observed.

To obtain further information, computed tomography angiography was performed, which showed significant enlargement of the main pulmonary artery. Although the right and left pulmonary arteries appeared normal at the hilum, their diameters at the ostium were suggestive of aneurysmal changes, and clear dilation of the main pulmonary trunk was noted (Figure 2).

The combination of severe pulmonary regurgitation and a PAA, together with the risk of aneurysm rupture, indicated an emergent clinical scenario necessitating urgent surgical intervention.

## Management

After induction of general anesthesia, a median sternotomy was performed. The pericardium was opened and suspended with stay sutures. To provide a cleaner and safer operative field, the patient was placed on CPB for a short duration (perfusion time: 29 minutes).

Given the patient's young age, the procedure being limited to the right side of the heart, the clear RV outflow tract anatomy, absence of severe adhesions or distortion, and no ventricular septal defect, the surgery was carried out on a beating heart. This approach was chosen to avoid RV injury from prolonged cross-clamping and cardioplegic arrest, thereby better preserving RV function. However, in patients with significant pulmonary hypertension or marked dilation, the beating-heart approach may limit surgical exposure, and in the event of bleeding or hemodynamic instability, conversion to an on-pump arrested-heart procedure may be required.

**Figure 3:**
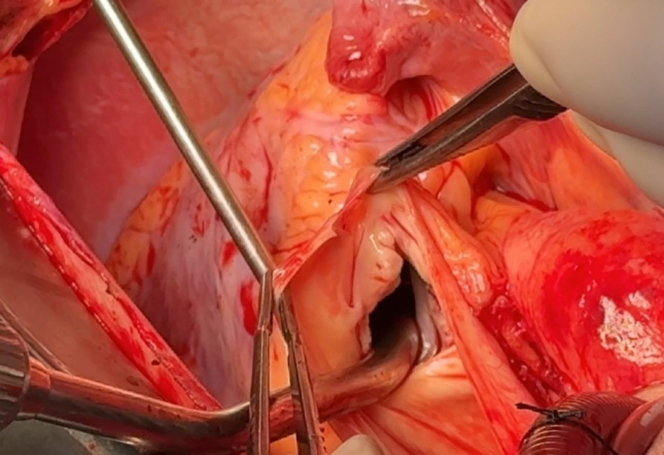
Intraoperative View of the Monocuspid Pulmonary Valve The valve is isolated and congenital, demonstrating a single-cusp morphology. Surgical inspection confirms the structural anomaly. Also shown in Video 1.

**Figure 4:**
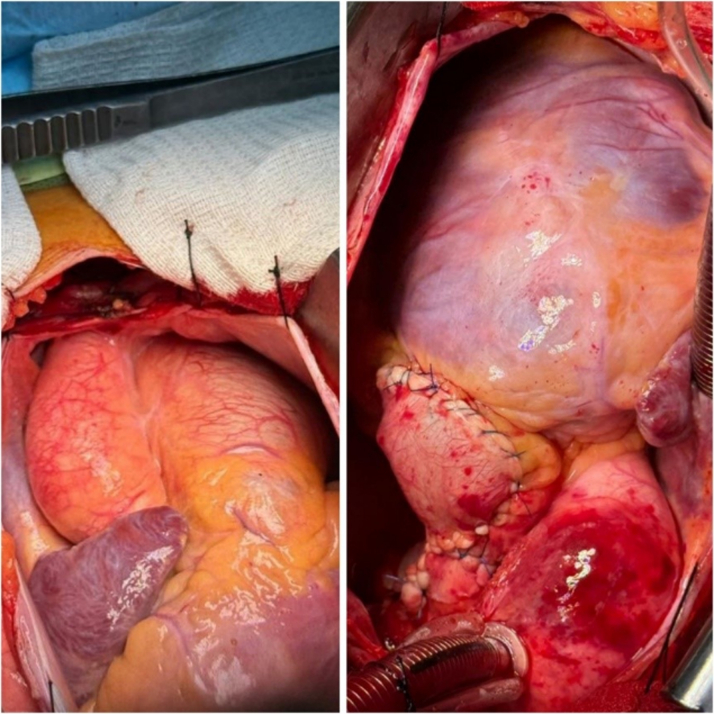
Intraoperative View Showing Completed Aneurysmorrhaphy, Summarizing the Surgical Procedure and Anatomical Repair The pulmonary trunk and both the right and left pulmonary arteries have been reconstructed, with suture lines clearly visible.

Intraoperatively, the pulmonary valve was observed to be monocuspid (Figure 3, Video 1). Subsequently, we performed aneurysmectomy and aneurysmorrhaphy on the main pulmonary artery and both right and left branches, followed by replacement with a synthetic graft (Figure 4). Pulmonary valve reconstruction at the annulus level was performed with running suture. The prosthesis size was determined intraoperatively according to annular measurement and RV outflow tract morphology. Ultimately, a 27-mm bioprosthesis provided the best fit, without tension or distortion. This choice ensured annular stability and also created an adequate landing zone for potential future interventions, such as valve-in-valve procedures (Video 2).

The pulmonary artery is a low-pressure system; autologous pulmonary tissue was used. No wrapping technique or artificial patch was applied in order to avoid the additional risks associated with prosthetic materials (Video 3). Finally, the sternum was closed in layers, and the patient was transferred to the intensive care unit in stable condition.

## Outcome and follow-up

Postoperative echocardiography showed a bioprosthesis with normal leaflet motion and no paravalvular or transvalvular leak, with good flow in both branches of the pulmonary arteries. Pulmonary artery pressure decreased slightly from 27 to 25 mm Hg, while the pulmonary artery diameter measured 24 mm after surgery. The postoperative course was uneventful, and the patient was discharged after 7 days in stable condition.

## Discussion

A monocuspid pulmonary valve is a rare congenital malformation that, owing to the incomplete closure of the valve, leads to progressive RV volume overload and, if untreated, may result in pulmonary hypertension. The resulting volume overload promotes pulmonary artery dilation and aneurysm formation. Our patient had a congenitally monocuspid pulmonary valve, which led to severe pulmonary valve insufficiency and aneurysm formation of the main pulmonary artery and its branches, including the right and left pulmonary arteries. Almost all cases of congenital absence of the pulmonary valve cusp are associated with other congenital heart defects,^4^ such as tetralogy of Fallot. In this patient however, the absence of other congenital heart defects made this case noteworthy as a rare cardiac anomaly.

PAA is a rare and heterogeneous pathology, with an incidence of approximately 0.007%. It is most often located in the main pulmonary artery (89%) and sometimes also in the main branches (11%).^5^ The most commonly reported etiology of PAA is its association with congenital heart disease with a large left-to-right shunt and pulmonary artery hypertension.^6^ There is no definitive approach for PAA because of the paucity of information regarding long-term outcomes after medical or surgical intervention.^7^ To prevent further progression of severe pulmonary valve insufficiency and potentially fatal complications such as pulmonary artery rupture, we performed resection and reconstruction of the aneurysmal segments. Although conventional CPB with cardioplegic arrest is associated with risks such as stroke, coagulopathy, and myocardial or respiratory dysfunction, in this case we performed CPB without inducing cardiac arrest. The heart was maintained in a beating state throughout the procedure, without cross-clamping and without cardioplegia. Furthermore, the short CPB duration helped maintain pressure, flow, and RV function within normal ranges and prevented potential RV injury even after surgery. This technique in itself represents a novel and noteworthy approach in the management of such cases.

## Conclusions

The monocuspid pulmonary valve is a congenital anomaly that may occur in isolation, without associated other structural cardiac defects. Progressive pulmonary valve insufficiency can lead to aneurysmal dilation of the pulmonary arteries. Early identification of such conditions can help prevent life-threatening complications such as dissection or rupture. Another important educational point in this case is the performance of surgery using the beating-heart technique, which is considered less invasive compared with traditional methods. Documenting this approach may enhance current clinical knowledge.

## Funding Support and Author Disclosures

The authors have reported that they have no relationships relevant to the contents of this paper to disclose.

## Appendix

Video 1 The Surgeon Elevates the Valve Leaflet to Demonstrate the Abnormal Anatomy and Monocuspid Morphology of the Pulmonary Valve This intraoperative finding confirmed the preoperative diagnosis and highlights its role in causing severe valvular regurgitation and right ventricular dilation in this patient.

Video 2 During Beating-Heart Surgery, the Surgical Team Is Shown Sizing the Pulmonary Valve Annulus With Prosthetic Sizers to Determine the Appropriate Prosthesis Size This step is crucial to ensure proper fitting, prevent paravalvular leak, and preserve right ventricular function.

Video 3 . Intraoperative View Showing the Surgeon Placing Sequential Sutures Along the Pulmonary Valve Annulus At this stage, the RVOT is opened to allow stable positioning of the prosthesis. Finally, autologous pulmonary tissue is used to cover the valve.
